# Laser microdissection of conifer stem tissues: Isolation and analysis of high quality RNA, terpene synthase enzyme activity and terpenoid metabolites from resin ducts and cambial zone tissue of white spruce (*Picea glauca*)

**DOI:** 10.1186/1471-2229-10-106

**Published:** 2010-06-12

**Authors:** Eric Abbott, Dawn Hall, Björn Hamberger, Jörg Bohlmann

**Affiliations:** 1 Michael Smith Laboratories, University of British Columbia, 2185 East Mall, Vancouver, B.C., V6T 1Z4, Canada; 2 Department of Botany, University of British Columbia, 6270 University Boulevard, Vancouver, B.C., V6T 1Z4, Canada

## Abstract

**Background:**

Laser microdissection (LMD) has been established for isolation of individual tissue types from herbaceous plants. However, there are few reports of cell- and tissue-specific analysis in woody perennials. While microdissected tissues are commonly analyzed for gene expression, reports of protein, enzyme activity and metabolite analysis are limited due in part to an inability to amplify these molecules. Conifer stem tissues are organized in regular patterns with xylem, phloem and cortex development controlled by the activity of the cambial zone (CZ). Defense responses of conifer stems against insects and pathogens involve increased accumulation of terpenoids in cortical resin ducts (CRDs) and *de novo *formation of traumatic resin ducts from CZ initials. These tissues are difficult to isolate for tissue-specific molecular and biochemical characterization and are thus good targets for application of LMD.

**Results:**

We describe robust methods for isolation of individual tissue-types from white spruce (*Picea glauca*) stems for analysis of RNA, enzyme activity and metabolites. A tangential cryosectioning approach was important for obtaining large quantities of CRD and CZ tissues using LMD. We report differential expression of genes involved in terpenoid metabolism between CRD and CZ tissues and in response to methyl jasmonate (MeJA). Transcript levels of β-pinene synthase and levopimaradiene/abietadiene synthase were constitutively higher in CRDs, but induction was stronger in CZ in response to MeJA. 3-Carene synthase was more strongly induced in CRDs compared to CZ. A differential induction pattern was observed for 1-deoxyxyulose-5-phosphate synthase, which was up-regulated in CRDs and down-regulated in CZ. We identified terpene synthase enzyme activity in CZ protein extracts and terpenoid metabolites in both CRD and CZ tissues.

**Conclusions:**

Methods are described that allow for analysis of RNA, enzyme activity and terpenoid metabolites in individual tissues isolated by LMD from woody conifer stems. Patterns of gene expression are demonstrated in specific tissues that may be masked in analysis of heterogenous samples. Combined analysis of transcripts, proteins and metabolites of individual tissues will facilitate future characterization of complex processes of woody plant development, including periodic stem growth and dormancy, cell specialization, and defense and may be applied widely to other plant species.

## Background

Complex metabolic processes in plants are often localized to specialized cells or tissues. The woody stem of a conifer contains a large number of specialized tissues that are organized in a regular pattern. The outer bark tissue (phloem, cortex and periderm) and the inner wood tissue (xylem) are separated by the cambial zone (CZ) [1]. Initial cells within the CZ give rise to sieve cells, parenchyma cells and fibers towards the phloem and parenchyma cells and tracheids towards the xylem. In spruce species (*Picea spp*.), large cortical resin ducts (CRDs) in the bark carry terpene-rich oleoresin that plays a role in defense against biotic stress such as insect feeding, egg deposition, or pathogen inoculation [2,3]. In response to biotic stress, tracheid mother cells in the CZ are transiently reprogrammed to produce additional traumatic resin ducts before resuming tracheid production, which is associated with increased defense and resistance [4,5]. Treatment of spruce stems with methyl jasmonate (MeJA) has been shown to elicit a response that mimics the response to biotic stress [6,7].

A number of different methods have been developed to isolate and enrich individual cell- or tissue-types from plants. In conifers, which include the economically important spruce and pine (*Pinus spp*.) species, and in other tree species such as poplars, enriched cell populations from stem tissues can be obtained by separating bark from wood [6,8], taking xylem scrapings [9,10] and by tangential cryosectioning across the CZ [11-13]. Other methods that have been applied in herbaceous plant species include isolation of glandular trichomes or epidermal cells from plant surfaces by abrasion [14,15] and generation of protoplasts for fluorescence activated cell sorting [16]. However, these latter methods would be difficult, if not impossible to apply for the isolation of specific cell- or tissue-types from the inner parts of woody stems of perennial species.

Laser microdissection (LMD) is a specific form of laser-assisted microdissection that uses a UV cutting laser to isolate tissues of interest from thin sections of biological samples, which are collected by gravity below the sample. LMD and other forms of laser-assisted microdissection are being applied widely in both animal and plant research [17,18]. The most common application of laser-assisted microdissection is for RNA isolation and transcript analysis by qRT-PCR and more recently by sequencing using high-throughput technologies [19]. Protein, enzyme and metabolite analysis has been limited partly because amplification is not possible for these molecules. Microdissected tissues have been successfully analyzed using proteomics [20] and metabolomics techniques [21], but there are few reports (none in plants) of isolation of intact protein samples for enzyme assays [22,23]. LMD has recently been applied successfully for microchemical analysis of stone cells from Norway spruce (*P. abies*) stems [24], but laser-assisted microdissection has not been widely applied to woody plant tissues. Among recent reports of combined analysis of RNA, protein and metabolites from individual cell-types, at least one study demonstrated the feasibility of combined transcript and metabolite analysis from laser microdissected samples [25-27].

In woody perennials, laser-assisted microdissection has the potential to further improve the degree of spatial resolution of sample dissection for the study of dynamic, tissue-specific processes. For example, the CZ controls several processes of interest including the periodically alternating events of stem growth and dormancy, wood development and induced defense of traumatic resin duct formation. In CRDs, we are interested in a better understanding of tissue- and cell-type specific processes of constitutive and possibly induced defense.

In this paper, we report the successful use of LMD technology for the isolation of individual specialized tissues from white spruce (*P. glauca*) stems suitable for subsequent combined analysis of RNA transcript abundance, enzyme activity and metabolite profiles. In validation of these combined methods, we show that genes involved in terpenoid biosynthesis and defense exhibit differential gene expression patterns between CRD and CZ tissues and in response to methyl jasmonate (MeJA) treatment. We further demonstrate that active terpene synthase (TPS) enzyme and terpenoid metabolites can be detected and analyzed in laser microdissected CRD and CZ tissues. The methods for LMD combined with analysis of gene expression, enzyme activity and metabolite profiles in microdissected samples will enable a more comprehensive analysis of complex metabolic processes at multiple levels of regulation in individual tissues that are otherwise difficult to access in woody plants.

## Results and Discussion

### Application of LMD technology to spruce stems

To maximize the number of cells harvested from a sample we required the ability to cut large regions from relatively thick tissue sections, which is possible using the LMD system as collection is aided by gravity. Plant tissue is inherently resistant to laser cutting due to the presence of cell walls, which can be highly lignified in spruce stem tissues. LMD was not effective in cutting spruce stem tissue mounted on glass slides because the laser power required was sufficient to etch the surface of a glass polyethylene naphthalate (PEN)-membrane slide resulting in diffusion of the beam and decreased laser cutting efficacy. However, the gravity assisted collection method of LMD permits the use of glass-free, steel frame polyethylene terephthalate (PET)-membrane slides that eliminates the need for glass support, facilitates the use of increased laser power and also allows collection of microdissected tissues in an empty PCR tube cap, which is required for metabolite extractions. LMD was used successfully to cut large areas (>1 mm diameter) from thick sections (>30 μm) mounted on PET-membrane frame slides. For routine LMD applications, we used sections of 25 μm. The LMD platform using PET-membrane frame slides was determined to be a highly suitable system for microdissection of spruce stem tissue.

### Overview of LMD from spruce stem samples

**Figure 1:**
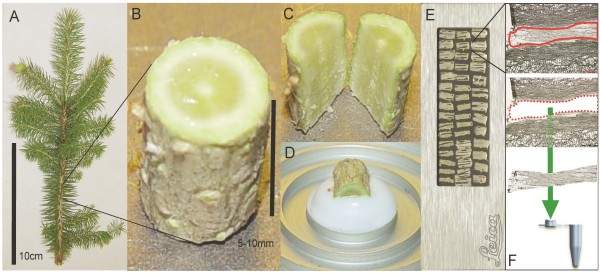
**Schematic overview of sample preparation for laser microdissection of spruce stems**. (A) 2-year old white spruce harvested above the root. (B) Stems were divided into 5 - 10 mm long pieces that were flash frozen in liquid nitrogen. (C) Stem pieces were split in half longitudinally prior to cryosectioning. (D) Half stem segment mounted for tangential cryosectioning. (E) Tangential cryosections on a PET-membrane frame slide. (F) Laser microdissection: Regions of interest are selected, cut with a laser and selected cells fall by gravity into a collection tube.

Sample preparation protocols for LMD can vary substantially depending on the type of tissue and downstream analysis. We describe here a single method of microdissecting two different specialized tissues from white spruce stems that is suitable for analysis of RNA, protein and metabolites (Figure 1). Briefly, frozen stem sections are taken from 2-year old spruce trees for cryosectioning in the tangential plane. Cryosections are mounted on a membrane support and selected tissues are isolated using LMD.

### Preparing tangential cryosections for LMD

Prior to sectioning, specimens are often fixed and embedded to preserve delicate cell structures [17,18,28]. However, fixation can be time consuming, non-uniformly reduces the extractability of molecules from the tissue sample [17,29] and can be a source of contamination [21]. We found that the morphology of formalin fixed, paraffin embedded cross-sections of spruce stems was of good quality, but the RNA was degraded compared to unfixed samples. Sucrose was tested as a cryoprotectant to preserve morphology but was found to interfere with laser cutting. Cryosections without cryoprotection or fixation showed reduced quality of morphology, but were still of high enough quality to identify specialized cell types and tissues and gave higher RNA yield and integrity. Therefore, cryosections were taken from unfixed, frozen stem pieces in either cross-section or tangential section orientation. Cryosections were then transferred onto a PET-membrane frame slide containing 100% ethanol (for RNA extraction) or DTT (for protein or metabolite extraction) and allowed to dry thoroughly.

**Figure 2:**
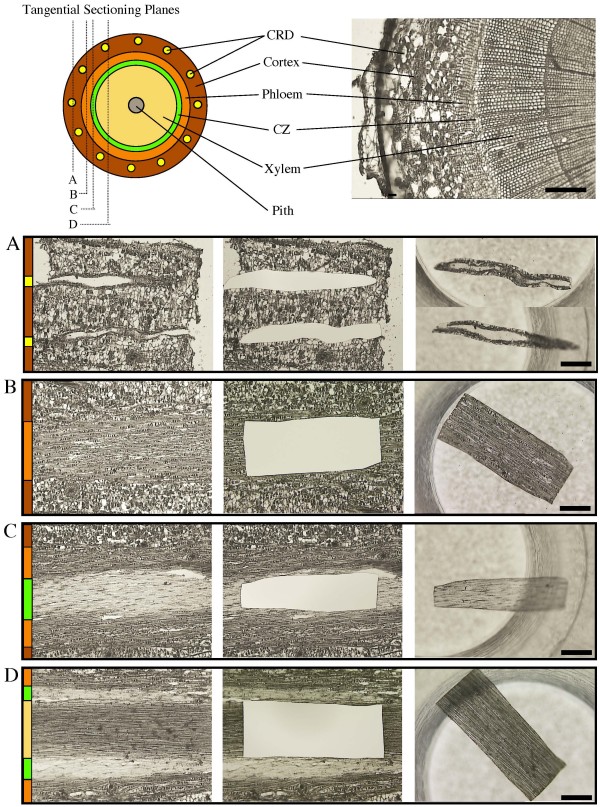
**Identification and microdissection of individual tissue types from tangential cryosections**. (Top) Schematic diagram and image of a stem cross-section showing an overview of tissue types. Letters A, B, C and D in the schematic indicate tangential cryosection planes corresponding to the figure panels (A), (B), (C) and (D) below. (A-D) Tissue images of tangential cryosections at different stem depths. For each horizontal figure panel (A) - (D), the left image shows tissue cryosection prior to laser cutting; the centre image shows the same tissue cryosection after laser cutting; and the right image shows the laser microdissected tissues in collection tube. Tissue types are indicated by the color bar on the left side of panels (A) - (D) where colors correspond to the colors in the schematic of the cross section (Top): Brown is cortex, yellow is cortical resin duct (CRD), orange is phloem, green is cambium zone (CZ), yellow is xylem, and grey is pith. (A) Tangential cryosection containing cortex and CRD tissues. (B) Tangential cryosection containing cortex and phloem tissues. (C) Tangential cryosection containing cortex, phloem, and cambium zone tissues. (D) Tangential cryosection containing phloem, cambium zone, and xylem tissues. CRD: Cortical resin duct; CZ: Cambium zone. Length of scale bar is 200 μm for the cross-section and 400 μm for tangential panels (A) - (D).

**Figure 3:**
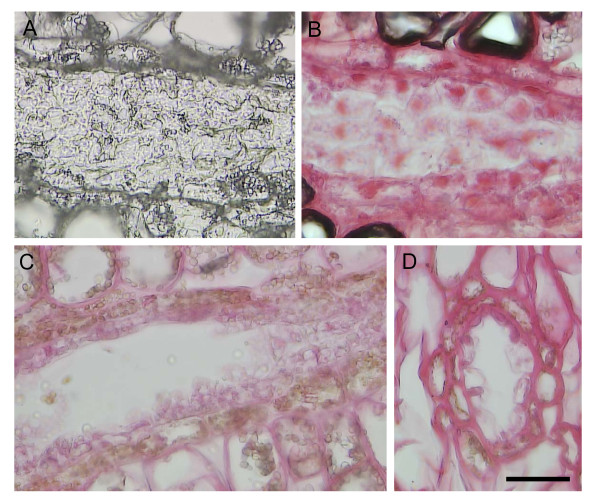
**Characterization of CRD morphology in cryosections before and after treatment with 100% ethanol**. (A) Tangential section of CRD treated with 100% ethanol. (B) Tangential section of CRD treated with 100% ethanol and stained with Safranin O. (C) Tangential section of CRD stained with Safranin O in 50% glycerol. (D) For orientation purposes, a cross-section of CRD and surrounding cortex tissue stained with Safranin O in 50% glycerol. Length of scale bar is 50 μm.

While stem cross-sections allow for the identification of many different tissue types within a single section (Figure 2, top), there is often damage to the cortex, CRD epithelia and CZ tissue, and these cryosections are prone to curling as they dry on the slide (not shown). In contrast, we found that tangential cryosections of woody stems are intact (Figure 2A-D, left panel) and do not curl on the slide unless there is a substantial amount of xylem tissue present. The quality of cell morphology was sufficient to identify major tissue types from tangential cryosections including CRDs, phloem, CZ and xylem (Figure 2A-D, middle and right panel). By carefully adjusting the sectioning plane, axially oriented tissue can be observed to extend the entire length of a tangential section (up to 10 mm long) compared to cross-sections where these same tissues may occupy only a small percentage of the section area. The morphology of tangential cryosections treated with 10 mM DTT was of similar quality but retained pigments such as chlorophyll in the outer stem tissues that are removed by ethanol treatment (not shown). Delicate tissues such as CZ or CRD epithelia were intact even after treatment with 100% ethanol as demonstrated by subsequent staining for cellular contents in CRD epithelial cells (Figure 3A and 3B). The quality of the morphology of ethanol dried cryosections was similar that cryosections mounted in 50% glycerol (Figure 3C).

A tangential sectioning orientation is well suited for LMD of spruce stems because morphology is well preserved without cryoprotection or fixation and larger regions of individual tissues are accessible compared to cross-sections, thus requiring fewer cryosections to obtain a large quantity of cells using LMD. Tangential cryosections with similarly intact morphology have also been successfully produced from white spruce needles (data not shown) suggesting that these techniques may be successfully applied to a broad range of cell- and tissue-types from large or small specimens.

### LMD of CRD and CZ tissues from tangential cryosections of spruce stems

A series of tangential cryosections from the cortex to the xylem was prepared and mounted on a single LMD frame slide. CRD and CZ tissues were chosen to test LMD applications and subsequent RNA, protein and metabolite analysis because they consist of metabolically active cells that represent a very small proportion of the spruce stem and are thus good candidates for high resolution enrichment using LMD. CRDs carry terpene-rich oleoresin and terpene synthase enzymes have been shown to be localized to CRD epithelial cells [30]. The CZ contains initial cells for differentiation of all secondary xylem and secondary phloem tissue and thus plays a vital role in stem growth and development as well as the formation of traumatic resin ducts.

Laser microdissected CRD and CZ tissues were collected separately into an empty PCR tube cap and tissues were checked for the quality of morphology after laser cutting (Figure 2A-D, middle and right panel). CRD tissue included the epithelial cells immediately lining the resin duct lumen as well as the second cell layer (Figure 2A, left panel and Figure 3). CZ tissue included all thin-walled, light colored cells that could be visually distinguished from fully differentiated xylem and phloem (Figure 2C-D, left panel). Laser settings were the same for cryosections treated with ethanol or DTT. Slides were mounted on the LMD system with tissue sections dried on the bottom surface to prevent microdissected cells from becoming trapped on top of the membrane after cutting. Laser cutting was very efficient with ~90% of microdissected regions being released from surrounding tissue and immediately falling into collection tubes. Regions that did not fall were dislodged using a laser pulse. To avoid cross contamination between different tissues cut from the same cryosections, each tissue type was harvested completely before selecting regions for the next tissue. Cryosections treated with DTT required a longer time to dry on the slide and sections that were not completely dried were difficult to cut with the laser.

The amount of tissue isolated using laser-assisted microdissection is often reported as the number of cells collected. However, cell size and metabolic state varies between different tissues and plant species and the total number of cells is difficult to estimate because the number of partially cut cells contained in thin cryosections depends on the section thickness. To facilitate better comparison of methods for laser-assisted microdissection we report the amount of microdissected tissue as "μl LMD volume", which is calculated as the microdissected area multiplied by the section thickness. The volume of CRD tissue that could be obtained by LMD from a given stem section of two year old white spruce trees was approximately threefold larger than the volume of CZ tissue. When calculated for a stem length of one centimeter, we obtained CRD with 2.9 ± 0.9 μl LMD volume/cm stem length (n = 7 biological replicates) compared to CZ tissue with 1.1 ± 0.3 μl LMD volume/cm stem length (n = 7).

### RNA extraction from CRD and CZ tissues isolated by LMD

Total RNA was extracted from CRD and CZ tissue isolated by LMD from ethanol treated cryosections obtained from a 6 mm long half stem segment. Cryosections were dried in 100% ethanol and microdissected tissue was collected in RNA lysis solution for extraction. For each RNA extraction, the average LMD volume used was 0.72 ± 0.16 μl (n = 3) for CRD and 0.37 ± 0.12 μl (n = 3) for CZ, which was sufficient for use with a standard RNA isolation protocol rather than a modified protocol specifically designed for microdissected samples. RNA yield was normalized to the total LMD volume for each tissue type. CZ yielded 357 ± 58 ng RNA/μl LMD volume (n = 3), which was more than twice the yield from CRD tissue at 150 ± 12 ng RNA/μl LMD volume (n = 3). Incubation of samples at 42°C in lysis buffer prior to RNA extraction did not increase yields. RNA samples were DNase treated and concentrated by ethanol precipitation. There was no measurable loss of RNA during ethanol precipitation (not shown).

**Figure 4:**
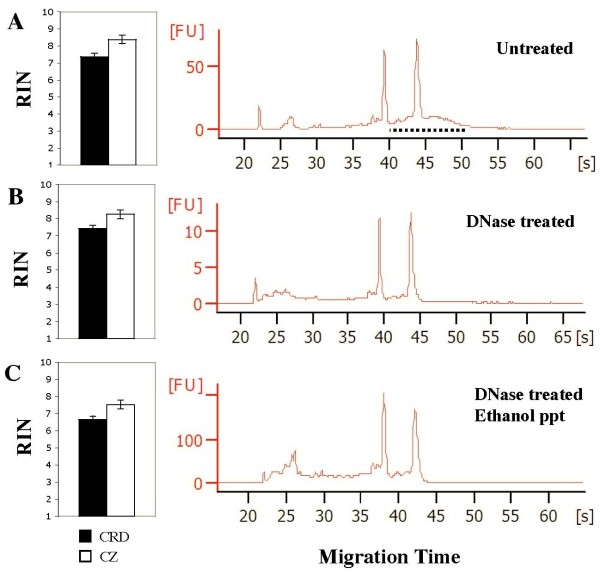
**RNA integrity of untreated, DNase treated and DNase/ethanol precipitated RNA samples from CRD and cambial zone tissue**. RNA integrity number (left) and a representative Bioanalyzer electropherogram (right) is shown for each treatment. (A) Untreated, (B) DNase treated, (C) DNase treated and ethanol precipitated RNA samples. Error bars represent standard error of three biological replicates. The dotted line in untreated samples (A) represents a region of genomic DNA background contamination that is removed by DNase treatment (B and C). RIN: RNA integrity number; FU: Fluorescence units.

RNA integrity was assessed using the Bioanalyzer RNA Pico Assay and expressed as an RNA integrity number (RIN). RNA yield was quantified using the Ribogreen assay, which was found to be robust with no interference from buffer components. High integrity RNA suitable for qRT-PCR analysis and construction of cDNA libraries was obtained from microdissected tissues with CZ RNA being of slightly higher quality than CRD RNA (Figure 4). RNA samples with an RIN greater than 5.0 are suitable for qRT-PCR [31] and an RIN greater than 7.0 is the standard for construction of cDNA libraries [Personal communication, Yungjun Zhao, BC Cancer Agency Genome Sciences Centre, Vancouver, BC]. DNase treatment eliminated background genomic DNA contamination (Figure 4) without decreasing RNA integrity, whereas ethanol precipitation resulted in a decrease of 0.7 RIN units. RNA integrity is of particular concern during LMD because each slide may be at room temperature for over an hour while regions of interest are selected and cut. To assess RNA degradation during this time, whole cross-sections were dried in ethanol on a PET-membrane frame slide and collected in RNA lysis buffer immediately or after incubation at room temperature. There was no detectable decrease in RNA integrity for slides left at room temperature for up to four hours (data not shown). Slides were also stored at -80°C for eight days with no decrease in RNA integrity (data not shown). However, cryosections may fall off the slide during storage and slides must be stored in a dry, airtight container to prevent condensation when they are warmed to room temperature.

To test if RNA integrity can be affected by LMD we applied extensive laser cutting to whole cross-sections but only observed a slightly lower RNA integrity (<1.0 RIN units) compared to RNA extractions from intact whole cross-sections. We found that RNA integrity from CRD and CZ tissue after microdissection, DNase treatment and ethanol precipitation is of sufficient quality for downstream applications. It was not necessary to apply any specific treatments to remove polysaccharides or polyphenols from LMD tissue and the use of the Plant RNA Isolation Aid (Ambion, USA) to remove these compounds was found to substantially reduce RNA yields. These results suggest that polysaccharides or polyphenols may not be abundant in CRD and CZ tissue or that these compounds may have been efficiently removed during sample preparation and RNA extraction.

### Transcript analysis from CRD and CZ tissue isolated by LMD

**Figure 5:**
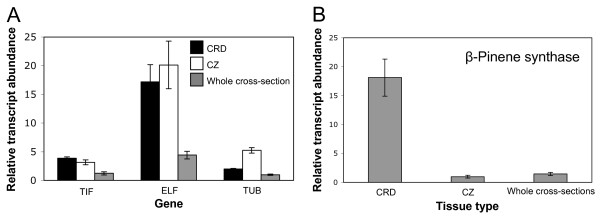
**Validation of reference genes and tissue-specific analysis of β-pinene synthase expression between CRD, CZ and whole cross-section tissues**. (A) Relative mRNA abundance of candidate reference genes normalized to RNA concentration. TIF: Translation initiation factor; ELF: Elongation factor; TUB: Tubulin α-subunit. (B) Relative transcript abundance of β-pinene synthase in different tissue types relative to TIF expression. Error bars represent the standard error of three biological replicates.

**Figure 6:**
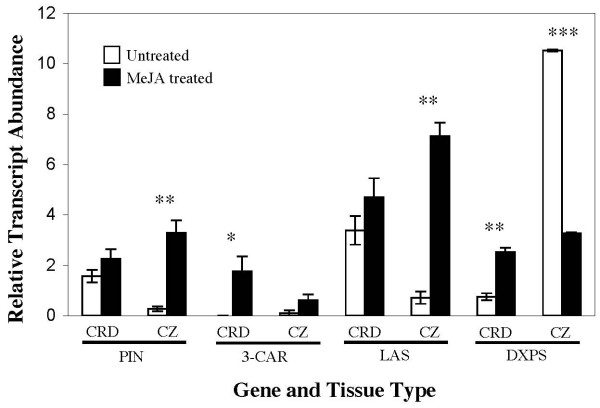
**Relative transcript abundance of selected terpenoid biosynthetic genes**. PIN: β-pinene synthase; 3-CAR: 3-carene synthase; LAS: levopimaradiene/abietadiene synthase; DXPS: 1-deoxyxyulose-5-phosphate synthase. Each gene is normalized to TIF expression. Error bars are the standard error of three biological replicates for all CRD and MeJA treated CZ but was restricted to two biological replicates for untreated CZ due poor template quality for one sample and limited quantity of biological material. Statistical significance represents a Student's T-test comparison of MeJA treated versus untreated samples with (*) p < 0.05, (**) p < 0.01 and (***) p < 0.001.

qRT-PCR is the most common method of quantitative transcript analysis. When transcript abundance is reported as a normalized value relative to a reference gene it is critical to carefully evaluate the reference gene to ensure that it is expressed at constant levels under experimental conditions being tested. We evaluated three candidate reference genes, translation initiation factor (TIF), elongation factor (ELF) and tubulin α-subunit (TUB), for expression in spruce stem CRD, CZ and whole cross-sections (Figure 5A). TIF was the most appropriate reference gene because it had the lowest standard deviation across the tissue types tested. Primers specific to a white spruce TPS gene (β-pinene synthase) [GenBank:BT105745] were used to compare relative transcript abundance for a representative monoterpene synthase gene involved in terpenoid defense metabolism [32] between CRD, CZ and whole cross-section tissues by qRT-PCR. β-Pinene synthase transcripts were 12-fold more abundant in CRD tissue, the primary site for constitutive terpenoid accumulation, compared to CZ and whole cross-section tissues (Figure 5B). Similarly, transcripts for levopimaradiene/abietadiene synthase (LAS) representing a major diterpene synthase for diterpene resin acid biosynthesis was more abundant in CRD than in CZ in untreated trees (Figure 6).

MeJA-inducible changes in transcript abundance were evaluated for four different genes involved in terpenoid biosynthesis, including two monoterpene synthases, a diterpene synthase, and one gene encoding 1-deoxyxyulose-5-phosphate synthase (DXPS) (Figure 6). CRD and CZ tissue were harvested eight days after MeJA treatment as this timepoint has been shown in Norway spruce to be the peak of TPS transcript abundance [32]. The TPS genes tested showed unique spatial expression patterns in response to MeJA treatment. In CZ tissue, expression of β-pinene synthase and LAS was up-regulated in response to MeJA treatment, but induction was not significant for 3-carene synthase. TPS induction in CZ tissue was likely associated with localized traumatic resin duct formation. In CRD tissue, a slight up-regulation was observed for β-pinene synthase and LAS reanscripts, but this induction is not significant and there were already high levels of transcripts for these genes prior to MeJA treatment. However, a significant induction was observed for 3-carene synthase in CRD tissue where constitutive expression is low for this TPS gene. Since CRDs are preformed prior to biotic stress they are also sometimes referred to as constitutive resin ducts [6,33,34]. The MeJA-inducible change of gene expression in CRD tissue indicates that these constitutively formed anatomical defense structures can be activated at the molecular level by MeJA, and possibly other stress. We have recently confirmed this result of CRD activation in independent work with Sitka spruce (*P. sitchensis*) by immunofluorescence localization of TPS protein [K. Zulak and J. Bohlmann, unpublished results].

DXPS transcript levels were also up-regulated in CRD tissue in response to MeJA treatment, but surprisingly DXPS transcript levels were down-regulated in CZ tissue in response to MeJA treatment. The particular DXPS gene tested here is 100% identical at the nucleotide level to a type II inducible DXPS from Norway spruce that was found to be up-regulated in the outer stem tissue (including bark and CZ tissue) in response to mechanical wounding, MeJA treatment and inoculation with fungal elicitors [35]. DXPS up-regulation in CRD tissue is consistent with a role in terpenoid oleoresin production. Down-regulation of DXPS transcripts in CZ tissue suggests subfunctionalization of this gene between these tissues. Since tracheid formation is transiently arrested during traumatic resin duct formation it is possible that this DXPS gene is involved in xylem development during constitutive growth. White spruce homologues have been identified for each of the three Norway spruce DXPS genes [35] and it is likely that one of these genes plays a role in terpenoid oleoresin production in developing traumatic resin duct tissue. In previous analysis of DXPS transcripts in Norway spruce [32,35], individual tissues were not separated and any detectable down-regulation of DXPS in CZ tissue may have been masked by the up-regulation of DXPS in CRD and other more abundant tissues. It is only by LMD isolation of CZ tissue from other tissues that DXPS down-regulation could be observed in the present study. Thus, LMD is an effective tool for elucidating tissue-specific gene expression patterns that is not possible using more conventional techniques of tissue separation.

### Protein extraction from CRD and CZ tissue isolated by LMD and detection of TPS enzyme activity in microdissected CZ tissue

Two-year old, MeJA treated white spruce trees used for protein extraction were harvested 8 days post-induction. This time point coincides with the peak of protein abundance for TPS enzymes in MeJA-induced Norway spruce [32]. For protein extractions, two stem pieces (13-15 mm total length) were taken from the apical end of the first interwhorl (previous year's growth) for LMD of CRD and CZ tissues from three independent biological replicates. The average LMD volume used for protein extraction was 5.14 ± 1.44 μl (n = 3) for CRD tissue and 1.44 ± 0.25 μl (n = 3) for CZ tissue. The protein extraction protocol was optimized to isolate active enzymes instead of applying higher yield protocols that use solvent extractions optimized for general proteomics analysis where active enzymes are not required. CZ yielded 31 ± 5 μg protein/μl LMD volume (n = 3), double the yield from CRD (16 ± 2 μg protein/μl LMD volume (n = 3)). A higher protein yield from CZ compared to CRD tissue correlates well with RNA yield from these tissues.

Monoterpene synthase assays were performed using approximately 30 μg total protein for CRD tissue, 15 μg for CZ tissue and 10 μg for whole cross-sections. The amount of protein used for each assay varied based on the total protein yield obtained from each tissue type. Monoterpene synthase enzyme activity was detected in assays with protein extracts from CZ samples and from whole cross-sections. Due to high levels of endogenous monoterpenes that were co-purified during the protein extraction, we could not detect monoterpene formation above background in assays with protein from CRD samples. However, monoterpene synthase enzyme activity was detectable in microdissected CZ tissue. The monoterpene synthase specific activity in MeJA-induced CZ tissue was 1.92 ± 0.31 pkat/mg total protein (n = 3), which was twice as large as in whole cross-sections (0.98 ± 0.29 pkat/mg total protein (n = 3)). The products of monoterpene synthase activity detected in CZ and whole cross-section extracts are shown in Table 1. Monoterpene synthase activity in the MeJA-induced CZ may be associated with the onset of traumatic resin duct development [6] and the differences in the product profiles of the monoterpene synthase activity in CZ tissue and whole cross-sections suggests differential expression of TPS gene family members in these tissues.

**Table 1:** Monoterpenes formed from geranyl diphosphate in cell-free enzyme assays of total protein extracts from CZ laser microdissected tissue and whole cross sections.

	Cambial Zone		Whole Cross Sections	
Product	% Total	St. Error	% Total	St. Error
(-)-α(-Pinene	22.2	1.0	3.9	0.8
(+)-α(-Pinene	19.0	0.8	2.9	0.5
(-)-β(-Phellandrene	28.4	0.7	12.3	3.7
(-)-β(-Pinene	20.7	1.0	8.4	0.3
(+)-3-Carene	8.1	1.2	25.6	2.2
(-)-Limonene	1.7	0.9	15.6	2.1
(α-Terpinolene	nd	nd	14.9	2.1
(+)-Sabinene	nd	nd	11.9	2.4
Myrcene	nd	nd	4.5	1.9

Treatment of cryosections with DTT was found to be critical to preserving enzyme activity. Using concentrations lower than 10 mM DTT or volumes lower than 2 μl resulted in browning of sections due to oxidation. Protein extractions from oxidized cryosections were degraded with smeared bands on silver stained SDS-PAGE gel (not shown) and no detectable monoterpene synthase enzyme activity. We did not observe a decrease in the level of specific monoterpene synthase enzyme activity due to laser cutting.

### Extraction and analysis of terpenoid metabolites in CRD and CZ tissue isolated by LMD

Sample preparation for metabolite extraction was similar to protein extraction. Tangential cryosections were taken from two-year old stems harvested 8 days after treatment with MeJA. Metabolite extractions were performed from a single stem piece (5-9 mm long) from the apical end of the first interwhorl of three independent biological replicates. The average LMD volume used for metabolite extraction was 2.68 ± 1.37 μl (n = 3) for CRD and 0.70 ± 0.25 μl (n = 3) for CZ. Microdissected tissues were transferred to a 2 ml glass gas chromatography vial using a pipette tip that had been dipped in water to reduce static. Metabolites were extracted in 500 μl methyl tert-butyl ether (MTBE) and split into two samples for independent analysis of mono- and diterpenes by gas chromatography-mass spectrometry (GC/MS).

**Figure 7:**
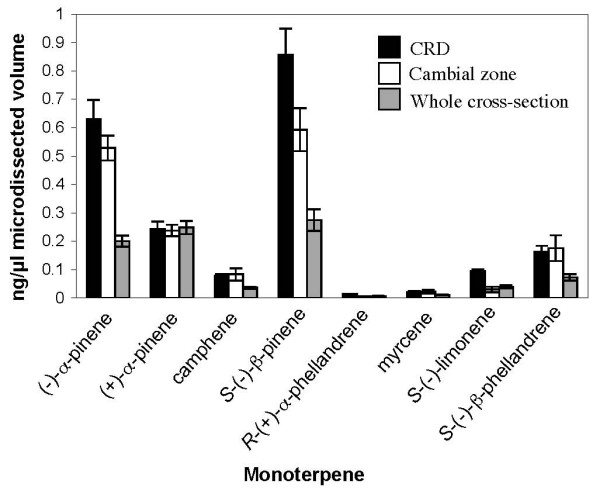
**Monoterpenes detected by GC/MS in metabolite extracts from CRD, CZ and whole cross-section tissue after MeJA treatment**. Only monoterpenes representing >1% total monoterpene content are shown. Error bars are the standard error of three biological replicates.

The total monoterpene yield was higher from CRD tissue with 2.39 ± 0.42 μg monoterpenes/μl LMD volume (n = 3) compared to 1.81 ± 0.41 μg monoterpenes/μl LMD volume (n = 3) from CZ tissue. This trend supports the observation that CRDs are the primary specialized tissues for oleoresin terpenoid accumulation in conifer stems [3]. The relative abundance of specific monoterpenes is similar between CRD, CZ and whole cross-section tissue, but the total monoterpene abundance is higher in the specialized tissues found in microdissected CRD and CZ samples (Figure 7). While this result is consistent with the general observation that terpenoids accumulate in CRDs and in the MeJA-induced traumatic resin ducts formed from initials in the CZ [3,6,7], to the best of our knowledge, these results are the first to specifically localize terpenoid profiles in these tissues. The most abundant monoterpenes detected from CZ tissue [(+)-α-pinene, (-)-α-pinene, (-)-β-pinene and (-)-β-phellandrene] correspond with the most abundant monoterpenes produced by TPS activity from GPP in cell free extracts isolated from this tissue (Table 1). However, the most abundant monoterpenes detected in whole cross-sections (including CRD tissue) do not correspond to the most abundant enzyme products. Monoterpenes are present in large quantities in the constitutive CRD oleoresin, so it is possible that newly induced TPS enzymes in this tissue may not have contributed substantially to the accumulated monoterpene composition at the time point measured.

Monoterpenes are volatile compounds and also may diffuse to some degree into the drop of 10 mM DTT during sample preparation. Therefore, it was necessary to evaluate the effect of sample processing on the terpenoid profile. Metabolite extractions were performed on cross sections immediately after sectioning or after drying on a drop of 10 mM DTT. As expected, the total monoterpene yield was lower in dried samples, which can be attributed to monoterpene volatility (Additional File 1: Figure S1A), but the relative abundance of individual monoterpenes was not changed substantially (Additional File 1: Figure S1B). The extent of monoterpene diffusion was evaluated by placing a series of tangential cryosections on 4 μl drops of 10 mM DTT, pipetting up and down, and then collecting the aqueous phase and tangential sections for separate metabolite extractions. Approximately 2-3% of individual monoterpenes were found in the aqueous phase (Data not shown). This is an upper limit for monoterpene diffusion since cryosections are normally placed on a smaller drop of DTT and are not agitated by pipetting. Since volatility and diffusion do not substantially alter the relative monoterpene composition it is likely that the monoterpene profiles measured in microdissected tissues is representative of their relative abundance *in vivo*. However, due to the volatility of monoterpenes we caution that the measurement of absolute abundance of monoterpenes using these methods requires the additional use of internal standards.

We also tested the feasibility of a qualitative analysis of diterpenoids, the principal non-volatile component of conifer oleoresin [30], in CRD and CZ tissues isolated by LMD from stem cryosections. The diterpene compounds detected in laser microdissected CRD and CZ tissues were predominantly the diterpene resin acids abietic acid, dehydroabietic acid, isopimaric acid, levopimaric acid, neoabiatic acid, palustric acid, and sandaracopimaric acid, along with minor amounts of the corresponding diterpene aldehydes, alcohols and olefins.

## Conclusions

Laser-assisted microdissection has previously been applied in several herbaceous plant species, mostly for the analysis of RNA transcripts [17,18]. We describe methods for the isolation of individual tissue-types from the stem of white spruce, a woody perennial species that is particularly recalcitrant to tissue- and cell-type specific analysis using conventional techniques. Sample preparation is simple, robust and may be broadly applicable to RNA, protein and secondary metabolite analysis. The use of tangential cryosections was instrumental in obtaining sufficient quantities of microdissected tissues for protein and metabolite analysis, which is often challenging due to the inability to amplify these molecules. Microdissected tissue quantities were sufficient for RNA isolation using standard protocols designed for larger, non-microdissected samples and transcript analysis by qRT-PCR without RNA amplification. Terpenoid biosynthetic genes were shown to exhibit differential patterns of gene expression between CRD and CZ tissues and in response to MeJA treatment. The detection of TPS enzyme activity from laser microdissected tissues suggests that sample preparation and LMD do not interfere with sensitive downstream biochemical analyses. Successful extraction and quantification of volatile monoterpenes and detection of a range of diterpenoids suggests that metabolite analysis could also be amenable to different classes of metabolites.

The combined analysis of RNA, protein, enzyme activity and metabolite profiles from individual conifer stem tissues will be extremely powerful when combined with the use of genomics resources [36,37] and the application of transcriptomics [10,38], proteomics [32,39,40] and metabolite profiling [32]. Tissue-specific analysis supported by LMD has the potential to substantially improve our understanding of complex processes including cell differentiation and specialization associated with stem growth, wood development and the inducible formation and activation of defense-related structures such as resin ducts. The methods described here should be broadly applicable to both woody and herbaceous species and will be of particular value for those perennial woody systems where genomics resources are already available such as poplar [41,42] and grapevines [43,44].

## Materials and methods

### Plant material, methyl jasmonate (MeJA) treatment, and collection of stem samples

White spruce (*Picea glauca*) seedlings of the genotype PG653 were clonally propagated by somatic embryogenesis and provided by Dr. Krystyna Klimaszewska (Natural Resources Canada, Canadian Forest, Sainte-Foy, Québec). Two-year old trees were maintained outside at the University of British Columbia greenhouse and moved, prior to experiments, into controlled greenhouse environments as previously described [8]. Trees were used for experiments at the beginning of the third year growth season and were actively growing with flushing buds present. Untreated trees for RNA extractions were harvested on April 27, 2009. MeJA treated trees for RNA, protein and metabolite extraction were induced on June 1, 2009 by applying a fine spray of 100 ml of 0.1% MeJA (Aldrich, USA) in 0.1% Tween 20 (Fisher Scientific, USA) over the entire stem as described previously [8] and samples were harvested eight days post-induction. Trees were cut at the base and the lateral branches and basal-most 2 cm of the stem were removed and discarded. The remaining stem was divided into pieces of 6 to 8 mm length using a thin razor blade (Wilkinson Sword, Classic), immediately flash frozen in liquid nitrogen and individually stored in 1.5 ml microcentrifuge tubes at -80°C.

### Cryosectioning

An individual stem piece was transferred to a Leica model CM3050 cryostat (Leica Microsystems, Germany) and allowed to equilibrate in the cryostat chamber (chamber temperature -15°C and object temperature -25°C) (Figure 1B). For subsequent LMD applications, the stem piece was divided in half along the longitudinal axis using a thin razor blade (Wilkinson Sword, Classic) (Figure 1C). Care was taken to ensure that any stem defects or buds were oriented away from the cryosectioning surface of the specimen. Each half stem segment was quickly and gently placed flat side down onto a small drop of Optimal Cutting Temperature embedding medium (Sakura Finetek USA, Inc., USA) (Figure 1D). Tangential cryosections of 25 μm thickness were taken with the stem axis oriented vertically during sectioning. Cryosections were immediately transferred to either a pool of cold 100% ethanol for RNA extractions or a 2 μl drop of 10 mM DTT for protein and metabolite extractions on a PET-membrane frame slide (#11505151, Leica Microsystems, Germany). Cryosections for morphological characterization were stained with 0.05% Safranin O in 50% glycerol. For other applications not involving LMD, the stem was mounted upright and four to eight cross sections, each of 25 μm thickness, were taken and transferred to a PET-membrane frame slide as for microdissected samples and collected using forceps.

### Laser microdissection (LMD)

Cryosections on PET-membrane frame slides were allowed to dry at room temperature before microdissection using a Leica model LMD6000 Laser Microdissection Microscope (Leica Microsystems, Germany) with 5× magnification, laser intensity between 110-128 and speed of 2. Laser microdissected CRD or CZ tissues were collected into the caps of nuclease free 0.5 ml PCR tubes (Axygen, USA) containing the appropriate buffer for RNA and protein extractions (described below) or into empty caps for metabolite extractions. Buffer crystallization on the LMD collection tube holder was reduced by adding buffer to the cap before mounting it on the holder. When microdissecting multiple tissues from a single cryosection, dissection of one tissue type was completed before starting to cut the next tissue type to avoid cross contamination of tissue samples. The LMD volume for a given sample collection was calculated by multiplying the section thickness by the total area of all microdissected regions for each tissue. Standard deviations are reported for LMD volume.

### RNA extractions

RNA was extracted independently from three separate trees using the standard-volume protocol (non-LCM) for the RNAqueous-Micro RNA Isolation Kit (Ambion, USA). Briefly, laser microdissected tissues were collected in 30 μl of RNA lysis solution in a 0.5 ml PCR tube cap and stored at -80°C. PCR tubes were thawed upside down on ice and microdissected tissues were transferred with lysis solution to a nuclease free 1.5 ml microcentrifuge tube containing an additional 30 μl lysis buffer. The PCR cap was washed twice with 20 μl lysis solution to give a total volume of 100 μl lysate for a silica column-based purification according to the manufacturer's protocol with elution of total RNA using 3 × 20 μl elution buffer heated to 75°C. DNase treatment was performed with reagents provided as described in the manufacturer's protocol. RNA samples were concentrated by ethanol precipitation by adding 0.1 volumes of DEPC treated 3 M sodium acetate and 2.5 volumes of cold 100% ethanol before freezing at -80°C for at least 30 minutes. RNA pellets were collected by centrifugation (18,000 × *g*, 30 min, 4°C), supernatant was removed by pipetting and pellets were washed with 100 μl of 70% ethanol made with DEPC-treated water. Centrifugation (18,000 × *g*, 10 min, 4°C) and removal of supernatant was repeated and the pellet was dried at room temperature for 10 min before resuspension in 10 μl DEPC-treated water. RNA quality was assessed using the RNA Pico Assay for the 2100 Bioanalyzer (Agilent, USA). RNA yield was determined by two replicate measurements using the Ribogreen assay (Invitrogen, USA). Standard deviation is reported for RNA yield in order to represent the variation of the extraction protocol.

### Quantitative Real Time PCR (qRT-PCR)

RNA from three independent trees was reverse transcribed in separate reactions using random hexamers and Superscript III reverse transcriptase (Invitrogen, USA). Gene targets were translation initiation factor (TIF), elongation factor (ELF), tubulin α-subunit (TUB), β-pinene synthase (PIN), 3-carene synthase (3CAR), levopimaradiene/abietadiene synthase (LAS) and 1-deoxyxyulose-5-phosphate synthase (DXPS) with the following sequences: ELF-f GTTGCTGTAACAAGATGGATGC; ELF-r CCCTCAAAACCAGAGATAGGC; TIF-f CATCCGCAAGAACGGCTACATC; TIF-r GTAACATGAGGGACATCGCAG; TUB-f TATGATGCCCAGTGATACGTCG; TUB-r ATGGAAGAGCTGCCGGTATGC; PIN-f CTACAAGGCGGACAGAGCC; PIN-r TGATCATGGCGTTGATATGGTC; 3CAR-f GGCTCTCCGTAGACCAACCTCAACTG; 3CAR-r GCACAAACAATATCTCTCCCAGGTCCAATG; LAS-f GGACGATCTCAAGTTGTTTTCCGATTC; LAS-r TGAGAACCACTGTTCCCAGCGC; DXPS-f AGAAACTCCCTGTGAGATTTGCCCTT; DXPS-r CAACAGTAACTGATATGCCCTGCTGAG. qRT-PCR reactions were performed in 96-well plate (HSP9655, BioRad, USA) sealed with a plastic film (MSB1001, BioRad, USA) using a DNA Engine Opticon 2 (MJ Research, USA) as follows: *Reaction mix: *0.1 μl cDNA (1.5-14 ng/μl), 3.65 μl DEPC-treated water, 3.75 μl pre-mixed primers (1.2 μM each), 0.03 μl HK-UNG thermolabile uracil N-glycosylase (Epicentre Biotechnologies, USA) and 7.5 μl DyNAmo HS SYBR Green qPCR 2x master mix (Finnzymes, Finland). *PCR program: *30 minutes at 37°C, 15 minutes at 95°C followed by 45 cycles of (10 s at 94°C, 30 s at 56°C, 30 s at 72°C followed by measurement of reaction fluorescence) and a 10 minute final extension at 72°C. A melting curve was generated from 65°C to 95°C at 0.2°C intervals holding each temperature for 1 s before measuring reaction fluorescence. Data was analyzed using Real Time PCR Miner [45]. Relative transcript abundance was calculated using the equation 1/(1+E)^CT ^where the cycle threshold (CT) is the average of four technical PCR replicates and the efficiency (E) is the average of all reactions across all templates for each primer set. Relative transcript abundance was then normalized to TIF expression. Product identity and specificity were confirmed by sequencing amplicons from representative reactions. Reactions with non-discrete melting curves or other anomalies were excluded from analysis.

### Protein extractions

Protein was extracted independently from three separate trees treated with MeJA. For each tree, microdissected tissue from two stem pieces (~16 mm combined length) was collected in four 0.5 ml PCR tube caps each containing 30 μl protein extraction buffer (50 mM HEPES pH 7.2, 5 mM DTT, 5 mM ascorbic acid, 5 mM sodium bisulfite, 10 mM MgCl_2_, 10% glycerol, 1% polyvinylpyrrolidone (PVPP), 0.1% Tween 20) and stored at -80°C. Samples from each tree were thawed upside down on ice and contents were pooled into a 1.5 ml microfuge tube and the PCR tube caps were each washed with 20 μl protein extraction buffer for a total volume of 200 μl. This volume was then supplemented with PVPP (1% w/v) and the protease cocktail described in Lippert et al., (2009) [40] modified by adding 10 mM 1,10-phenanthroline (Sigma, USA), 0.5 mM PMSF (Sigma, USA) and by using 1 μM E64 and 0.5 mM AEBSF. The sample was then ground by hand with a microtube pellet pestle (Kontes, USA) for 1 min, gently vortexed intermittently for 1 min and sonicated for 10 min in a 4°C water bath sonicator (Model 1510, Branson, USA). Homogenized samples were centrifuged at 120 × *g *for 10 min at 4°C and 130 μl of supernatant was desalted using a PD25 spintrap desalting column (#28-9180-04, GE Healthcare, USA) equilibrated with metal ion free desalting buffer (25 mM HEPES pH 7.2, 100 mM KCl, 10% glycerol). Protein concentration was determined by Bradford assay. Standard deviation is reported for protein yield in order to represent the variation of the extraction protocol.

### Monoterpene synthase enzyme assays

Enzyme assays were performed on protein samples from three separate MeJA-treated trees based on previously published methods developed for large volume biological samples [6,46,47] with minor modifications for laser microdissected tissue samples. Briefly, 40-55 μl cell free total protein extract with a protein content of 10-30 μg was combined with geranyl pyrophosphate (GPP, Echelon Biosciences Inc., USA) and monoterpene synthase buffer (25 mM HEPES pH 7.2, 100 mM KCl, 10 mM MnCl_2_, 10% glycerol, 5 mM DTT) to a total volume of 500 μl and a final substrate concentration of 50 μM in a 2 ml GC vial. As a control, protein samples were boiled for 10 min prior to assay. The aqueous assay mixture was overlaid with 0.5 ml pentane containing 2.5 μM isobutyl benzene as an internal standard and incubated at 30°C for 100 minutes. Assays were vortexed for 20 s and immediately stored for at least 30 min at -80°C before centrifugation (1000 × *g*, 4°C, 30 min) and GC/MS analysis as described below. Standard error is reported for enzyme activity.

### Metabolite extractions

Terpenoid metabolites were extracted from three separate MeJA-treated trees based on previously published methods [6,48] with the following modifications for laser microdissected tissues. Microdissected tissues from a single stem piece (~8 mm length) were collected in an empty 0.5 ml PCR tube cap and transferred to a GC vial (#5183-2072, Agilent, USA) using a wet pipette tip (to reduce static) and stored at -80°C until extraction. Metabolites were extracted in 500 μl MTBE containing 3.0 μM isobutyl benzene as an internal standard and shaken vigorously overnight at room temperature. For monoterpene analysis, 400 μl of MTBE extract was combined with 150 μl of 0.1 M ammonium carbonate (pH 8) and vortexed for 20 s before carefully transferring the top ether layer to a new GC vial insert for GC/MS analysis. For diterpene analysis, 100 μl of MTBE extract was combined with 40 μl methanol and 40 μl TMS-diazomethane and incubated for 45-60 min at room temperature. Samples for diterpene analysis were dried under high purity grade nitrogen and resuspended in 100 μl diethyl ether (inhibitor free, HPLC grade) and transferred to a fresh GC vial insert for GC/MS analysis. Standard error is reported for metabolite yields in order to represent the variation of the extraction protocol.

### Metabolite analysis by gas chromatography-mass spectrometry (GC/MS)

The protocol for GC/MS analysis of mono- and diterpenoids was based on previously published methods [6,48]. Metabolites in 1 or 2 μl pentane (extracted from enzyme assays) or MTBE (extracted directly from tissue) were identified using a GC (Agilent 6890A series) coupled with a mass spectrometer (5973N mass selective detector, quadropole analyzer, electron ionization, 70 eV). Metabolite identification was based on comparison of retention times to authentic standards as well as comparison to mass spectral libraries (Wiley7Nist05). Monoterpenes were separated using a DB-WAX capillary column (J&W 122-7032, 0.25 mm diameter, 30 m length, 0.25 μm film thickness) with the following program: 4 min at 40°C, increase by 3°C/min to 85°C, increase by 30°C/min to 250°C, hold for 2.5 min (Injector = 250°C, initial flow rate = 1.4 ml He/min, total run time 27.00 min). Stereochemistry of monoterpenes was determined for compounds where authentic standards were available by separation on a Cyclodex-B chiral capillary column (J&W 112-2532, 0.25 mm diameter, 30 m length, 0.25 μm film thickness) with the following program: 1 min at 55°C, increase by 1°C/min to 100°C, increase by 10°C/min to 230°C, hold for 10 min (Injector = 250°C, initial flow rate = 1.0 ml He/min, total run time 69.00 min). Diterpene compounds were separated using an AT-1000 capillary column (Alltech A-13783, 0.25 mm diameter, 30 m length, 0.25 μm film thickness) with the following program: 1 min at 150°C, increase by 1.5°C/min to 220°C, increase by 20°C/min to 240°C, hold for 15 min (Injector = 250°C, initial flow rate = 1.0 ml He/min, total run time 63.67 min).

## Authors' contributions

EA, DH and JB designed experiments, conducted the data analysis and interpretation of data and results. EA and DH carried out experiments. BH contributed to LMD method development. EA, DH, and JB wrote the manuscript. All authors read and approved the final manuscript.

## Supplementary Material

Additional file 1 **Figure S1 - Effects of drying whole cross-sections in a drop of 10 mM DTT on monoterpene yield and relative monoterpene profile**. (A) Monoterpene yield; (B) Relative monoterpene profile. Only monoterpenes representing >1% total monoterpene yield are shown. Error bars represent the standard error of three biological replicates. Click here for file
