# Supplementary figures and images for: Laser microdissection of conifer stem tissues: Isolation and analysis of high quality RNA, terpene synthase enzyme activity and terpenoid metabolites from resin ducts and cambial zone tissue of white spruce (Picea glauca)
Source: BMC Plant Biol. 2010 Jun 12;10:106. doi: 10.1186/1471-2229-10-106 (PMC3095273; doi:10.1186/1471-2229-10-106)

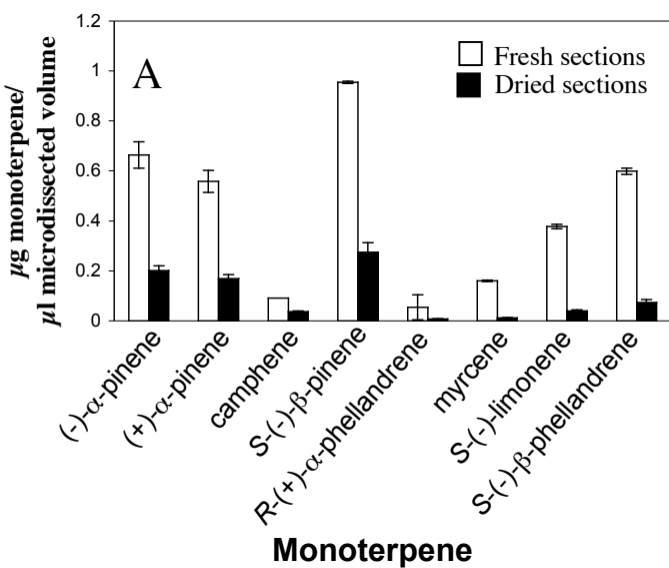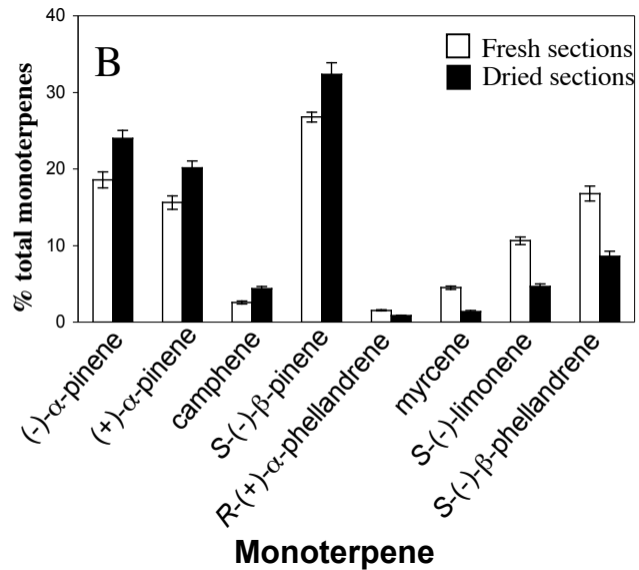

Supplement: Additional file 1 — Figure S1 - Effects of drying whole cross-sections in a drop of 10 mM DTT on monoterpene yield and relative monoterpene profile. (A) Monoterpene yield; (B) Relative monoterpene profile. Only monoterpenes representing >1% total monoterpene yield are shown. Error bars represent the standard error of three biological replicates. [file 1471-2229-10-106-S1.PDF]
